# Hyperinsulinemia Enhances Hepatic Expression of the Fatty Acid Transporter Cd36 and Provokes Hepatosteatosis and Hepatic Insulin Resistance*

**DOI:** 10.1074/jbc.M115.640292

**Published:** 2015-06-17

**Authors:** Pär Steneberg, Alexandros G. Sykaras, Fredrik Backlund, Jurate Straseviciene, Ingegerd Söderström, Helena Edlund

**Affiliations:** From the ‡ Umeå Centre for Molecular Medicine and; the § Department of Public Health and Clinical Medicine, Umeå University, SE-901 87 Umeå, Sweden

**Keywords:** fatty acid transport, hepatocyte, insulin, insulin resistance, Type 2 diabetes, CD36, PPARγ, fatty liver

## Abstract

Hepatosteatosis is associated with the development of both hepatic insulin resistance and Type 2 diabetes. Hepatic expression of Cd36, a fatty acid transporter, is enhanced in obese and diabetic murine models and human nonalcoholic fatty liver disease, and thus it correlates with hyperinsulinemia, steatosis, and insulin resistance. Here, we have explored the effect of hyperinsulinemia on hepatic Cd36 expression, development of hepatosteatosis, insulin resistance, and dysglycemia. A 3-week sucrose-enriched diet was sufficient to provoke hyperinsulinemia, hepatosteatosis, hepatic insulin resistance, and dysglycemia in CBA/J mice. The development of hepatic steatosis and insulin resistance in CBA/J mice on a sucrose-enriched diet was paralleled by increased hepatic expression of the transcription factor *Ppar*γ and its target gene *Cd36* whereas that of genes implicated in lipogenesis, fatty acid oxidation, and VLDL secretion was unaltered. Additionally, we demonstrate that insulin, in a *Ppar*γ-dependent manner, is sufficient to directly increase Cd36 expression in perfused livers and isolated hepatocytes. Mouse strains that display low insulin levels, *i.e.* C57BL6/J, and/or lack hepatic *Ppar*γ, *i.e.* C3H/HeN, do not develop hepatic steatosis, insulin resistance, or dysglycemia on a sucrose-enriched diet, suggesting that elevated insulin levels, via enhanced CD36 expression, provoke fatty liver development that in turn leads to hepatic insulin resistance and dysglycemia. Thus, our data provide evidence for a direct role for hyperinsulinemia in stimulating hepatic Cd36 expression and thus the development of hepatosteatosis, hepatic insulin resistance, and dysglycemia.

## Teaser

**Background:** Enhanced hepatic expression of the fatty acid transporter *Cd36* correlates with liver fat accumulation, hepatosteatosis, insulin resistance, and hyperinsulinemia.

**Results:** Insulin increases hepatic Cd36 expression in a *Ppar*γ-dependent manner.

**Conclusion:** Hyperinsulinemia stimulates hepatic Cd36 expression, which correlates with the development of hepatosteatosis, hepatic insulin resistance, and dysglycemia.

**Significance:** Hyperinsulinemia contributes to the development of hepatosteatosis and insulin resistance.

## Introduction

T2D^2^ is a current global epidemic, and similarly NAFLD, currently the most common chronic liver disease worldwide, is increasing globally with an estimated overall prevalence of ∼30% and with a prevalence up to ∼75% in obese individuals and Type 2 diabetics (1–3). NAFLD covers a spectrum of conditions associated with lipid accumulation in hepatocytes ranging from fatty liver, *i.e.* hepatosteatosis, to nonalcoholic steatohepatitis, advanced fibrosis, and cirrhosis (1, 3). Several independent factors, including (i) increased free fatty acid (FFA) uptake, (ii) *de novo* lipogenesis, (iii) decreased FA oxidation, and (iv) reduced VLDL secretion, may contribute to hepatic fat accumulation (2, 4, 5). The fatty acid transporter Cd36 (also known as FA translocase) functions as an important mediator of hepatic FA uptake (6). *Cd36* mRNA levels are drastically increased in livers of murine models of obesity and T2D (7), and *CD36* expression correlates with liver TG accumulation, insulin resistance, and hyperinsulinemia in human NAFLD (8, 9). Moreover, under normal, nonmetabolically challenged conditions forced expression of *Cd36* alone increases FA uptake in mouse hepatocytes *ex vivo* and mouse liver TG content *in vivo* (10). Nonetheless, the factors provoking increased hepatic *Cd36* expression remain unknown.

Hepatosteatosis is associated with development of hepatic insulin resistance and T2D and, vice versa, T2D is an established risk factor for the development of NAFLD (4, 11). Development of T2D is initially characterized by decreased insulin sensitivity, manifested by ensuing hyperglycemia and a compensatory response where pancreatic β-cells produce and secrete more insulin, which in turn results in hyperinsulinemia. Thus, hyperinsulinemia, which is associated with both T2D and NAFLD, is generally considered a consequence of insulin resistance. Accumulating data suggest, however, that hyperinsulinemia may actually drive and/or perpetuate insulin resistance (12, 13). Additionally, it has been reported that high insulin levels are positively correlated with the development of NAFLD in nondiabetic humans (14). We previously showed that *Gpr40*, also known as *Ffar1,* mediates high fat diet-induced hyperinsulinemia, and additionally our study suggested that hyperinsulinemia, hepatic *Cd36* expression, hepatosteatosis, and insulin resistance were associated (15). The expression of *Ppar*γ, an upstream regulator of *Cd36* expression (16, 17), has also been linked to hepatosteatosis. Although hepatic *PPAR*γ expression is relatively low under nonmetabolically stressed conditions (18), hepatic *Ppar*γ expression is increased in obese and diabetic mouse models (19) as well as in human NAFLD (8, 20), and hepatic overexpression of *Ppar*γ provokes fatty liver development in mice (21–25), whereas liver-specific inactivation of *Ppar*γ in *ob/ob* and AZIP mice reduces hepatosteatosis (26–28). Here, we provide evidence that hyperinsulinemia triggers fatty liver development and insulin resistance by stimulating hepatic expression of *Cd36*. A 3w sucrose-enriched diet was sufficient to provoke hyperinsulinemia, increased hepatic *Ppar*γ and Cd36 expression, hepatosteatosis, hepatic insulin resistance, and dysglycemia in CBA/J mice. We also show that high levels of insulin alone directly enhance Cd36 expression in perfused livers and isolated hepatocytes and that insulin stimulation of hepatic Cd36 expression is *Ppar*γ-dependent. Our data suggest a direct and sufficient role for hyperinsulinemia in *Ppar*γ-mediated enhancement of hepatic Cd36 expression and thus the subsequent development of hepatosteatosis.

## Experimental Procedures

### 

#### 

##### Animals

CBA/J mice were purchased from Scanbur, Denmark. C3H/HeN and C57BL/6J mice (stock number 000656, Jax mice) were purchased from Taconic. Male animals were used in all experiments. Animals were fed standard diet (CRM (E), Special Diet Service) and maintained on a 12:12-h light/dark cycle in a temperature/humidity-controlled (22 °C/50% humidity) room. For the sucrose-rich diet (SRD) regime, 10-week-old CBA/J, C57BL/6J, or C3H/HeN mice were given regular chow and 32% sucrose (Sigma) drinking water. The animal studies were approved by the Institutional Animal Care and Use Committee of Umeå University. Animal experiments were performed in accordance with the Guidelines for the Care and Use of Laboratory Animals.

##### Metabolic Parameters

Blood/plasma samples were collected from 6-h fasted CBA, B6, and C3H mice. Blood glucose was measured using glucometer (Ultra 2, One Touch), and plasma insulin was analyzed by an ultrasensitive mouse insulin ELISA kit (Crystal Chem Inc.). Glucose-stimulated insulin secretion (GSIS), glucose tolerance test (GTT), and insulin tolerance test (ITT) was performed as described in (15). Area under the curve was calculated according to the trapezoid rule. The homeostasis model for insulin resistance (HOMA-IR) was calculated from the fasting blood glucose (mmol/liter) × fasting plasma insulin (microunits/liter) divided by 22.5. Oil-red-O staining of liver sections and determination of liver triglycerides were performed as described previously (15).

##### In Vivo Insulin Signaling

To address hepatic insulin signaling, we performed intraperitoneal injections of insulin (0.1 milliunits/μl in 1× PBS) or equal volume control solution (1× PBS) in conscious mice (1.5 milliunits/kg). 20 min after injection, mice were killed, and livers were rapidly (within 30 s) dissected. Liver pieces were immediately put into liquid nitrogen and then stored at −80 °C for Western blot analysis.

##### Liver Perfusion Assay

Livers of 10-week-old male B6 or C3H mice were perfused with a 37 °C control (EBSS, E3024, Sigma, + 0.1% BSA) or insulin solution (10 nm insulin, Actrapid, in EBSS + 0.1% BSA) at flow rate of 100 ml/h by inserting a 27-gauge syringe in the portal vein and cutting open the right atrium of the heart. After 1 h, the liver was dissected into pieces that were immediately frozen in liquid nitrogen and stored at −80 °C for Western blot analysis.

##### Primary Hepatocyte Isolation, Culture, and Stimulation with Insulin

Primary hepatocytes were isolated from CBAxB6 F1 mice, following the protocol described previously (29). Isolated primary hepatocytes were seeded on 12-l or 6-well collagen I-coated plates (BD Biosciences) and cultured in a 37 °C/5% CO_2_ incubator. Primary hepatocytes were incubated for 4 h in DMEM supplemented with 10% (v/v) FCS, 1× GlutaMAX (Life Technologies, Inc.), and penicillin/streptomycin to promote cell adhesion to the wells, followed by a change of media to serum-free Medium 199 (Gibco, Life Technologies, Inc.) supplemented with 100 nm dexamethasone and penicillin/streptomycin, and incubated overnight. The hepatocytes were then exposed to fresh media with or without 10 nm insulin (Actrapid) and incubated for 5 and 45 min. At the end time point, the hepatocytes were harvested for Western blot analysis. In each biological replicate, the experiment for the 45-min time point was performed in duplicate/triplicate wells.

##### Western Blot

Liver tissue whole cell lysates containing an equal amount of protein were run on 4–15% polyacrylamide gels. For PKCϵ translocation experiment, 100 mg of liver tissue was used as starting material, and subcellular fractionation was performed with the subcellular protein fractionation kit for tissues (Pierce). Antibodies used for immunoblotting analysis are listed in Table 1. Semi-quantitative densitometry analysis of signals was performed using ImageJ software (National Institutes of Health). Quantification values were normalized against GAPDH (cytoplasmic fractions and whole liver lysates) or Na/K-ATPase (membrane proteins), and the relative mean protein expression ratio of control samples was arbitrarily set to 1. For PKCϵ translocation, the membrane (normalized to Na/K-ATPase)/cytosolic (normalized to GAPDH) fraction ratio was calculated (arbitrary units) (11), and the mean ratio of CD samples was arbitrarily set to 1.

**TABLE 1:** **Antibodies used in the study** Conditions refer to the buffer in which the antibody was diluted. TBST and PBST buffers contained 0.1% Tween 20.

Antigen	Species	Supplier	Dilution-conditions
Cd36	Goat	R&D Biosystems (AF2519)	1:1000 TBST + 5% BSA
Phospho-AKT(Ser^473^)	Rabbit	Cell Signaling (catalog no. 9271)	1:1000 TBST + 5% BSA
(pan)-AKT	Rabbit	Cell Signaling (catalog no. 4685)	1:1000 TBST + 5% BSA
GAPDH	Rabbit	Cell Signaling (catalog no. 2118)	1:50,000 TBST + 5% BSA
PKCϵ	Rabbit	Santa Cruz Biotechnology (C-15) (sc-214)	1:1000 TBST + 5% BSA
Na/K-ATPase	Rabbit	Cell Signaling (catalog no. 3010)	1:1000 TBST + 5% BSA
PPARγ	Mouse	Santa Cruz Biotechnology (E-8) (sc-7273)	1:1000 TBST + 5% BSA
Protein-disulfate isomerase	Rabbit	Abcam (catalog no. 3672)	1:1000 5% dry milk

##### qRT-PCR

qRT-PCR was performed as described previously (15), using as template the cDNAs prepared separately from each biological replicate. In all cases, reactions were run in duplicate and TATA-binding protein was used as a reference gene. The comparative threshold cycle method (Δ*Ct* method) was used to determine relative amounts of mRNAs in all samples. For all genes, relative mRNA expression in CD samples was arbitrarily set to 1. Primers used for qRT-PCR are listed in Table 2.

**TABLE 2:** **Oligomers used for qRT-PCR analysis** Targets in boldface represent human genes. Primers against TBP recognize both human and mouse gene.

Target	Forward primer (5 → 3)	Reverse primer (5 → 3)
*Tbp*	GAATTGTACCGCAGCTTCAAAA	AGTGCAATGGTCTTTAGGTCAAGTT
*Cd36*	TCATATTGTGCTTGCAAATCCAA	GCTTTACCAAAGATGTAGCCAGTGT
*PPAR*γ*2*	TGCCTATGAGCACTTCACAAGAAAT	CCGAAGTTGGTGGGCCAGAA
*PPAR*γ*1*	GCGGGCTGAGAAGTCACGTT	ACCGCTTCTTTCAAATCTTGTCTGTC
*Mogat1*	TCTACACTGTTGTTGGCCGC	CTGCTCTGAGGTCGGGTTCA
*Fabp4*	CCGCAGACGACAGGAAGGT	AGGGCCCCGCCATCT
*Fatp2*	GCTGACATCGTGGGACTGGT	TTCGACCCTCATGACCTGGC
*Fatp5*	ACCACTGGACTCCCAAAGCC	AGGACAGCACGTTGCTCACT
*Fabp5*	TCTTAAGGATCTCGAAGGGAA	CTTCCTAAGAGCCAGTCCTACT
*Chrebp*	ACTCAGGGAATACACGCCTACAG	TCTTGGTCTTAGGGTCTTCAGGAA
*Srebp1*	TGACCCGGCTATTCCGTGA	CTGGGCTGAGCAATACAGTTC
*Srebp1c*	TGCACATTTGAAGACATGCTC	AGCATAGGGGGCGTCAAACA
*Fasn*	GGAGTTCTCAGGCCGGGATA	GGGTACATCCCAGAGGAAGTCA
*Acc-1*	TGGGGATCTCTGGCTTACAGG	AGCCAGACATGCTGGATCTCAT
*Scd-1*	GGCCTGTACGGGATCATACTG	GGTCATGTAGTAGAAAATCCCGAAGA
*Elovl6*	AGCACCCGAACTAGGTGACA	TGGTGGTACCAGTGCAGGAA
*Cpt-1a*	GCAGACTCGGTCACCACTCA	GTGAACTGGAAGGCCACAGC
*Mttp*	TGCAGATGGACAAGGCTGAA	CCCTGCCTGTAGATAGCCTTTC
*Lpk*	GGCATCGAAAGTGGAAAGCT	GCCAGCCTGTCACCACAAT
***Cd36***	AGTTGGAACAGAGGCTGACAACT	TATGGGATGCAGCTGCCACAG
***PPAR*γ**	AAGGCGAGGGCGATCTTG	ATCATTAAGGAATTCATGTCGTAGATGAC

##### Cell Culture

Human liver hepatocellular carcinoma cell line HepG2 cells (ATCC-HB-8065) were maintained in a humidified incubator at 37 °C, 5% CO_2_. HepG2 cells were cultured in DMEM with 1 g/liter glucose (catalog no. 21885-025, Gibco), supplemented with 10% FBS (catalog no. 10500-064, Gibco), 1% nonessential amino acids (catalog no. 11140-035, Gibco), 1% penicillin/streptomycin (catalog no. 15070-063, Gibco), and 0.05% gentamicin (catalog no. 15750-037, Gibco).

##### siRNA Transfection

siRNA oligomers were purchased from Sigma. PPARγ siRNA sequence was obtained from Ref. 21. This siRNA recognizes a sequence that is conserved within all PPARγ isoforms in both mice and humans. MISSION siRNA universal negative control 1 (Sigma) was used as control siRNA. HepG2 cells and primary hepatocytes were transfected using Lipofectamine RNAiMax (Life Technologies, Inc.) according to the reverse transfection protocol. HepG2 cells were transfected with control and PPARγ siRNA (final concentration 10 nm) and were seeded in 24-well plates (50,000 cells/well). Primary hepatocytes were transfected with control and PPARγ siRNA (final concentration 10 nm) and were seeded in 12-well collagen-coated plates (100,000 cells/well). 24 h after transfection, cell culture medium was replaced with either control medium or medium containing 100 mm insulin, and cells were incubated for 2 h (HepG2 cells) or 100 min (primary hepatocytes). 30 h after transfection, HepG2 cells serum-containing culture medium was replaced with serum-free culture medium. 48 h after transfection, cell culture medium was replaced with control medium or medium containing 10 nm insulin, and cells were incubated for 2 h (HepG2 cells) or 100 min (primary hepatocytes). After this incubation period, cells were harvested for RNA preparation.

##### Statistical Analysis

All data are presented as mean ± S.E. In all experiments, *n* refers to the number of biological replicates. Data were analyzed and graphs were generated using Microsoft Excel and GraphPad Prism 6. A two-tailed Student's *t* test was used for all analyses between the two groups or the two treatments, and the following *p* values were considered to be statistically significant: *, *p* < 0.05; **, *p* < 0.01; ***, *p* < 0.001.

## Results

### 

#### 

##### CBA/J, but Not C57BL6/J, Mice on a Sucrose-rich Diet Become Hyperinsulinemic and Glucose-intolerant

**FIGURE 1.**
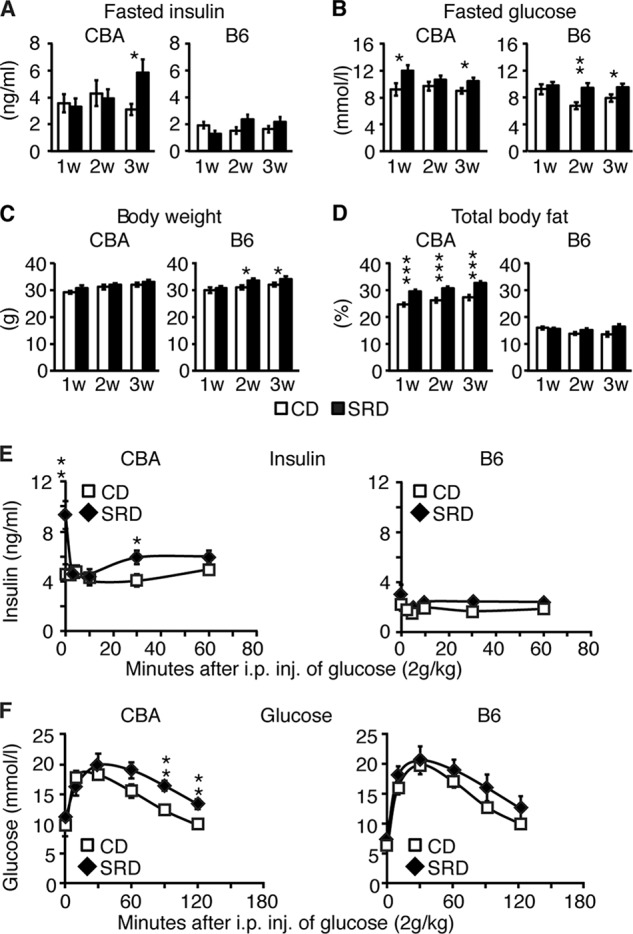
**CBA mice on SRD develop hyperinsulinemia and glucose intolerance.** *A* and *B*, fasted circulating insulin (*A*) and glucose (*B*) levels in CBA (CD *n* = 15 and SRD *n* = 15) and B6 (CD *n* = 5 and SRD *n* = 5) mice. *C* and *D*, body weights (*C*) and total body fat content (*D*) in CBA (CD *n* = 15–20 and SRD *n* = 15–20) and B6 (CD *n* = 8–15 and SRD *n* = 8–15) mice. *E* and *F*, insulin (*E*) and glucose (*F*) levels during GTT in 3w CBA (CD *n* = 10 and SRD *n* = 10) and B6 (CD *n* = 5 and SRD *n* = 5) mice. Data are presented as mean ± S.E. *, *p* < 0.05; **, *p* < 0.01; ***, *p* < 0.001; *inj,* injection.

To investigate a potential correlation between hyperinsulinemia and development of hepatosteatosis, we established an *in vivo* model for early onset hyperinsulinemia by exposing CBA/J (CBA), *i.e.* high insulin secreting, and C57BL6/J (B6), *i.e.* low insulin secreting, mice to a standard diet (CD) or standard diet plus 32% sucrose in the drinking water (sucrose-rich diet (SRD)) during a 3w period. Under these regimes, fasted insulin levels were significantly increased in 3w SRD CBA as compared with 3w CD CBA mice (Fig. 1*A*), whereas fasted insulin levels were overall lower in B6 mice and did not increase on a sucrose-rich diet, not even at 3w (Fig. 1*A*). Thus, a 3w SRD provokes early onset hyperinsulinemia in CBA but not B6 mice. In both CBA and B6 mice on SRD, the fasted blood glucose levels were slightly increased during the 3w period (Fig. 1*B*).

SRD B6 mice showed a modest increase in body weight at 2 and 3w as compared with age-matched CD B6 mice, although there was no difference in body weight between the CD and SRD group of CBA mice (Fig. 1*C*). Nonetheless, total body fat content was increased already after 1 week in SRD CBA mice compared with CD CBA mice (Fig. 1*D*). In contrast, total body fat content was generally lower in B6 mice and did not increase in response to SRD (Fig. 1*D*). GTT confirmed the hyperinsulinemic phenotype of 3w SRD CBA mice; thus, in addition to fasting hyperinsulinemia, GSIS was significantly increased at 30 min following glucose injection (Fig. 1*E*). Consistent with the development of hyperinsulinemia and enhanced GSIS, CBA mice on 3w of SRD were glucose-intolerant (Fig. 1*F*). In contrast, 3w SRD B6 mice did not show enhanced GSIS or glucose intolerance during a GTT (Fig. 1, *E* and *F*). Taken together, these data show that, regardless of diet, nonstimulated and stimulated insulin levels were markedly higher in CBA mice than in B6 mice. In addition, 3w of SRD provoked hyperinsulinemia, glucose intolerance, and increased body fat content in CBA mice. In contrast, B6 mice have lower basal levels of insulin compared with CBA mice and did not develop hyperinsulinemia nor glucose intolerance on increased body fat mass, in response to the 3w of SRD.

##### CBA Mice on SRD Develop Hepatosteatosis and Hepatic Insulin Resistance

**FIGURE 2.**
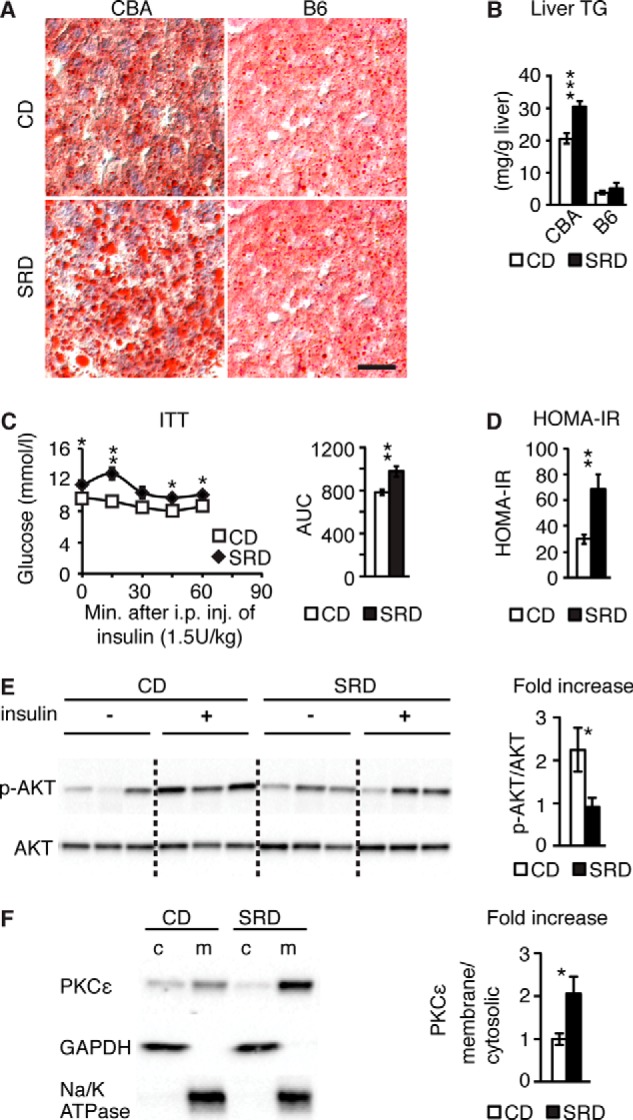
**3w SRD results in hepatic steatosis and insulin resistance in CBA mice.** *A,* representative Oil-red-O staining of fat droplets in liver sections from 3w and SRD CBA and B6 mice. *Scale bar,* 25 μm. *B,* TG content was measured in livers from 3w CBA (CD *n* = 10 and SRD *n* = 10) and B6 (CD *n* = 5 and SRD *n* = 5) mice. *C,* ITT in 3w CBA (CD *n* = 6 and SRD *n* = 6) mice. Diagram to the *right* show area under the curve (*AUC*) for the ITT. *D,* HOMA-IR in 3 weeks CBA (CD *n* = 20 and SRD *n* = 20) mice. *E,* representative Western blot and quantification of AKT phosphorylation in livers from 3w CD (*n* = 5) and SRD (*n* = 5) treated CBA mice following insulin injections. *Dotted lines* separate noncontiguous lanes of the same gel. *F,* PKCϵ membrane translocation assay. Liver lysates were fractionated from 3w CD (*n* = 4) and SRD (*n* = 4)-treated CBA mice. Representative Western blot and quantification of membrane (*m*)/cytosolic (*c*) ratio of PKCϵ are presented. The experiment was repeated with pooled liver samples (*n* = 6–7) with similar results. Data are presented as mean ± S.E., where *, *p* < 0.05; **, *p* < 0.01; ***, *p* < 0.001.

The hyperinsulinemia in 3w SRD CBA mice was paralleled by hepatic lipid accumulation as evidenced by increased accumulation of fat droplets and increased TG levels (Fig. 2, *A* and *B*), providing evidence of hepatosteatosis. In contrast, hepatic TG content was not increased in B6 mice following 3w of SRD (Fig. 2, *A* and *B*). Notably, the TG content of CD B6 livers were considerably lower than that of CD CBA mice, thus paralleling the overall lower basal insulin levels of B6 mice compared with CBA mice. Hepatic fat accumulation is frequently associated with hepatic insulin resistance (30), and the combined observation of hepatic steatosis, hyperinsulinemia, and glucose intolerance displayed by 3w SRD CBA mice suggested that they were insulin-resistant. ITT and homeostasis model assessment of insulin resistance (HOMA-IR) confirmed that 3w SRD CBA mice were insulin-resistant (Fig. 2, *C* and *D*).

Insulin stimulation of AKT phosphorylation (Ser^473^) was markedly reduced (by ∼50%) in livers of 3w SRD CBA as compared with 3w CD CBA mice (Fig. 2*E*), providing molecular evidence that the SRD provoked hepatic insulin resistance in these mice. During fatty liver development, pathological accumulation of diacylglycerol stimulates the activation of PKCϵ by enhancing its translocation to the plasma membrane, thereby impairing canonical insulin signaling that in turn leads to hepatic insulin resistance (11). We therefore next examined the subcellular localization of PKCϵ in livers of 3w SRD CBA mice and observed a 2-fold increase in the membrane/cytosolic PKCϵ ratio as compared with 3w CD CBA mice (Fig. 2*F*). Thus, 3w of SRD not only provoked hyperinsulinemia and hepatosteatosis in CBA mice but also resulted in development of hepatic insulin resistance, as illustrated by the reduced *in vivo* response to exogenous insulin during an ITT, increased HOMA-IR, reduced insulin-stimulated hepatic AKT phosphorylation, and increased hepatic PKCϵ membrane translocation. Taken together, these data link hyperinsulinemia, hepatic fat accumulation, and hepatic insulin resistance and suggest that increased insulin levels promote hepatic fat accumulation and thus hepatic insulin resistance.

##### Insulin Enhances Hepatic Cd36 Expression

**FIGURE 3.**
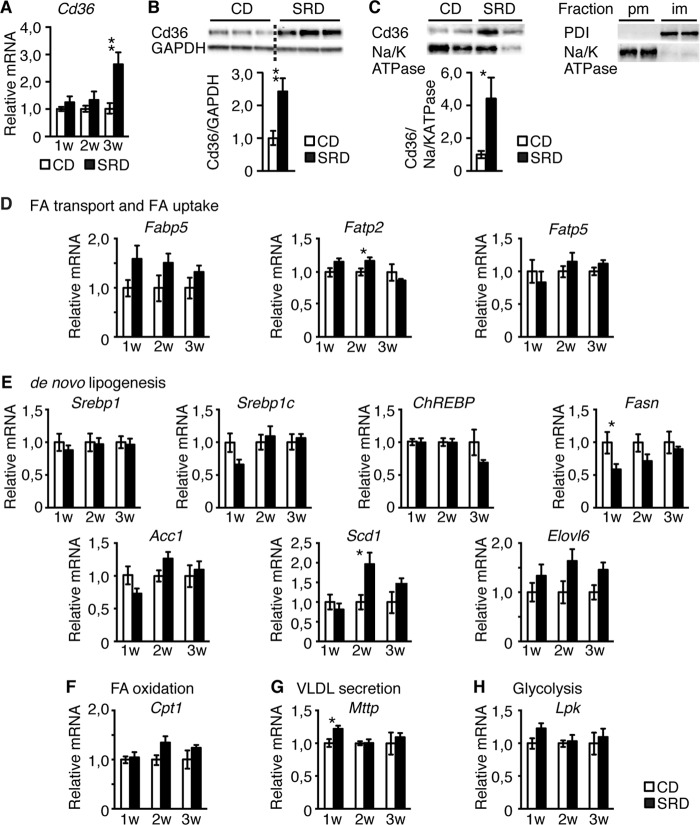
**Hepatic Cd36 expression is increased in 3w SRD CBA mice.** *A,* real time PCR analyses of hepatic *Cd36* expression in CD (*n* = 6) and SRD (*n* = 5–6)-treated CBA mice. *B,* representative Western blot and quantification of Cd36 expression in liver extracts isolated from CBA mice after 3 weeks of CD (*n* = 12) and SRD (*n* = 12). Each lane represents a biological replicate. *Dotted lines* separate noncontiguous lanes of the same gel. *C,* representative Western blot and quantification of Cd36 localization in hepatic plasma membrane fractions isolated from 3-week-treated CBA (CD *n* = 4 and SRD *n* = 4) mice. Control Western blot to the *right* show protein-disulfate isomerase (*PDI*) and Na/K-ATPase markers for plasma membrane (*pm*) and intracellular membrane (*im*) fractions, respectively, to confirm the purity of the prepared plasma membrane fractions. *D–H,* qRT-PCR expression analyses of indicated genes in livers from CBA mice CD (*n* = 6) and SRD (*n* = 5–6). Data are presented as mean ± S.E., where *, *p* < 0.05; **, *p* < 0.01.

To elucidate the molecular mechanisms underlying increased hepatic fat content in CBA mice on SRD, we assessed the expression of key components of FA uptake, FA trafficking, FA synthesis, and FA oxidative pathways. mRNA and protein levels of the FA transporter Cd36 were markedly increased in livers of CBA mice at 3w of SRD (Fig. 3, *A* and *B*), as was the plasma membrane localization of Cd36 (Fig. 3*C*), thus coinciding with the increase in insulin levels. In contrast, hepatic mRNA levels of the FA transporters *Fatp2* and *Fatp5* and the FA-binding protein *Fabp5* were largely unchanged in CBA mice at 3w of SRD (Fig. 3*D*). Expression of the key lipogenic transcription factors *Srebp-1, Srebp-1c* and *ChREBP*, which regulate FA biosynthesis, was also unchanged as was the expression of the key enzymes for lipogenesis as follows: *Fasn* and Acc1, FA desaturation; *Scd1*, and FA elongation; *Elovl6* (Fig. 3*E*). Moreover, the expression of the FA β-oxidation enzyme, *Cpt1a* (Fig. 3*F*), the rate-limiting enzyme of very low density lipoprotein (VLDL) secretion, *Mttp* (Fig. 3*G*), and the ChREBP target rate-limiting enzyme of glycolysis, *Lpk* (Fig. 3*H*), was unaltered in livers of CBA mice during the 3-week period of SRD. Thus, although we cannot exclude a role for *de novo* lipogenesis, reduced FA oxidation, or decreased VLDL secretion as contributors to the increased triglyceride content observed in CBA mice following 3w of SRD, our data imply a role for enhanced Cd36 expression in the development of hepatic steatosis in these mice.

**FIGURE 4.**
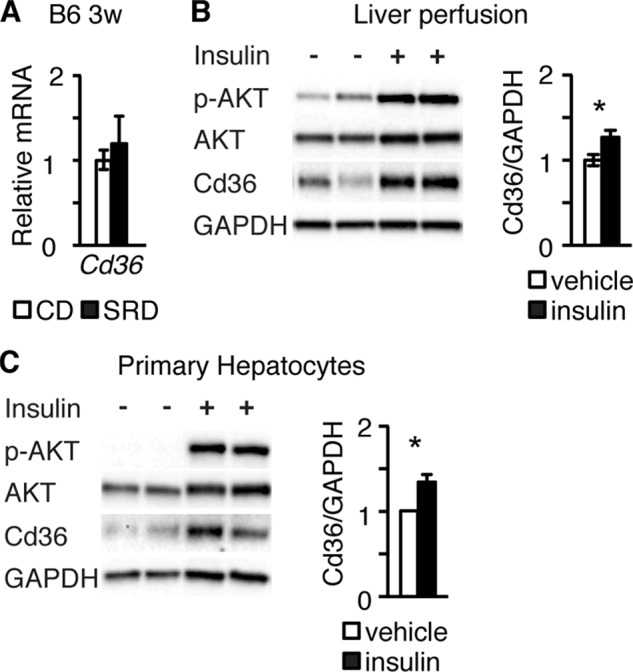
**Insulin enhances Cd36 expression in perfused livers and primary hepatocytes.** *A,* real time PCR analyses of hepatic expression *Cd36* in liver lysates isolated from B6 mice after 3 weeks on CD (*n* = 6) and SRD (*n* = 6). *B,* representative Western blot of phosphorylated AKT and Cd36 protein expression in total protein lysates from vehicle and insulin perfused B6 livers. Quantification of Cd36 expression in B6 vehicle (*n* = 12) and insulin (*n* = 9) perfused livers is shown in the diagram. *C,* representative Western blot of phosphorylated AKT and Cd36 expression in vehicle and insulin-cultured primary hepatocytes. Quantification of Cd36 expression assessed by Western blot densitometry is shown in the diagram (*n* = 5). Data are presented as mean ± S.E., where *, *p* < 0.05.

*Cd36* mRNA expression was not increased in livers of B6 mice at 3w of SRD (Fig. 4*A*), raising the notion that the elevated levels of insulin observed in 3w SRD CBA, but not B6 mice, increase hepatic Cd36 expression and thus likely hepatic FA uptake. If so, exposure of B6 livers to high insulin levels would result in enhanced Cd36 expression. To test this idea, we perfused livers of CD-fed B6 mice with 10 nm insulin. Perfusion of B6 livers with insulin not only resulted in robust Ser^473^ AKT phosphorylation (Fig. 4*B*), demonstrating that B6 livers were insulin responsive, but also in increased levels of Cd36 (Fig. 4*B*). Similarly, exposure of isolated primary mouse hepatocytes to 10 nm insulin also resulted in distinct Ser^473^ AKT phosphorylation and enhanced Cd36 levels (Fig. 4*C*). These results show that hyperinsulinemia not only correlates with enhanced hepatic Cd36 expression *in vivo* but that high levels of insulin are sufficient to directly increase hepatic Cd36 expression both in liver perfusion experiments and in cultured primary hepatocytes. Together, these findings provide evidence that hyperinsulinemia, by stimulating hepatic Cd36 expression, promotes hepatosteatosis *in vivo*.

##### Insulin-mediated Stimulation of Hepatic Cd36 Expression Is Pparγ-dependent

**FIGURE 5.**
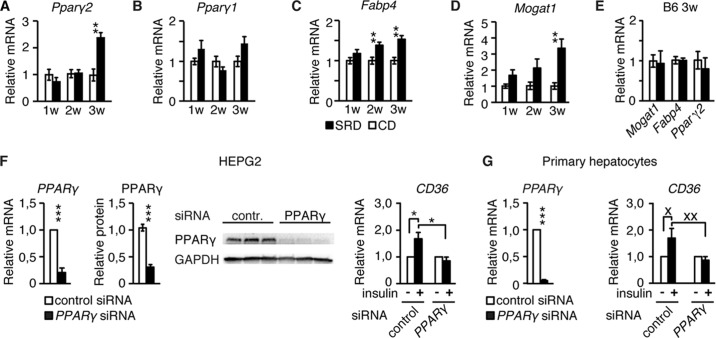
**Insulin-mediated enhancement of hepatic Cd36 expression is *PPAR*γ-dependent.** *A–E,* real time PCR analyses of *Ppar*γ*2* (*A*), *Ppar*γ*1* (*B*), *Fabp4* (*C*), and *Mogat1* (*D*) hepatic expression in CBA mice after 1–3 weeks of CD (*n* = 6) and SRD (*n* = 5–6) and in B6 mice (*E*) after 3 weeks of CD (*n* = 6) and SRD (*n* = 6). *F,* real time PCR and Western blot analyses of *PPAR*γ (in control and *PPAR*γ siRNA-transfected cells) and *CD36* expression in siRNA-treated HepG2 cells incubated with insulin (*n* = 6–7). *G,* real time PCR analyses of *Ppar*γ (in control and *Ppar*γ siRNA-transfected cells) and *Cd36* expression in siRNA-treated primary hepatocytes in the presence of absence of insulin (*n* = 9). Data are presented as mean ± S.E., where *, *p* < 0.05; **, *p* < 0.01; ***, *p* < 0.001. × represents *p* = 0.09, and ×× represents *p* = 0.06.

Several transcription factors, including Pparγ, PXR, LXR, and KLF2, have been implicated in the regulation of *Cd36* expression (16, 17, 31). Among these, the expression of the *Ppar*γ*2* isoform is not only up-regulated under hepatosteatosis conditions (18, 19) but also stimulated by insulin in primary hepatocytes (32). Consistent with these findings and in agreement with the increased expression of Cd36, hepatic *Ppar*γ*2* expression was up-regulated more than 2-fold in CBA, but not B6, mice on 3w SRD (compare Fig. 5, *A* and *E*), suggesting that hyperinsulinemia enhanced hepatic *Ppar*γ*2* expression in 3w SRD CBA mice. There was also a nonsignificant tendency of increased hepatic *Ppar*γ*1* expression in 3w SRD CBA mice as compared with 3w CD CBA mice (Fig. 5*B*). Alike *Cd36,* the expression of other Pparγ target genes, such as the intracellular *FA chaperone fatty acid-binding protein 4* (*Fabp4*) and *monoacylglycerol acetyltransferase* (*Mogat1*), which enhances hepatic fat accumulation by stimulating incorporation of FAs into TG via a FA biosynthesis-independent pathway (33, 34), was also increased in livers of CBA mice at 3w of SRD (Fig. 5, *C* and *D*), whereas hepatic *Mogat1* and *Fabp4* expression was unaltered in 3w SRD B6 mice (Fig. 5*E*). To directly investigate the role for *Ppar*γ in stimulation of hepatic Cd36 expression, we used siRNA to knock down *Ppar*γ in HepG2 cells and primary mouse hepatocytes. HepG2 cells and primary mouse hepatocytes in which *Ppar*γ expression had been knocked down failed to enhance *Cd36* expression in response to insulin (Fig. 5, *F* and *G*). Taken together, these results provide evidence that insulin enhancement of hepatic *Cd36* expression is *Ppar*γ-dependent.

**FIGURE 6.**
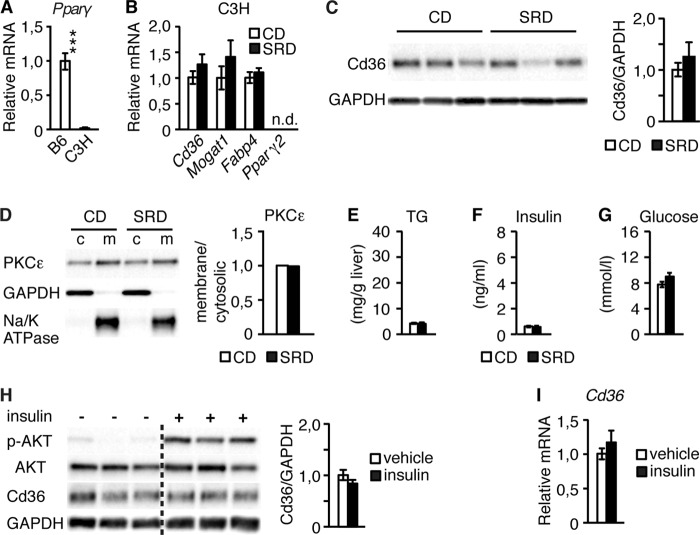
**Insulin fails to induce Cd36 expression in C3H livers.** *A* and *B*, real time PCR analyses of hepatic expression of *Ppar*γ (*A*) in liver extracts from B6 (*n* = 6) and C3H (*n* = 5) mice and *Cd36*, *Mogat1*, *Fabp4*, and *Ppar*γ*2* expression (*B*) in liver extracts from C3H mice after 3 weeks of CD (*n* = 4) and SRD (*n* = 5) are shown. *C,* representative Western blot and quantification of Cd36 expression in liver cell lysates from 3-week CD (*n* = 5) and SRD (*n* = 5) C3H mice. *D,* representative Western blot and quantification of membrane/cytosolic ratio of PKCϵ in liver extracts pooled from 3w CD (*n* = 5) and SRD (*n* = 5)-treated C3H mice separated into cytosolic (*c*) and membrane (*m*) fractions. *E–G*, hepatic TG (*E*), plasma insulin (*F*), and blood glucose (*G*) levels in C3H mice after 3 weeks of CD (*n* = 5) and SRD (*n* = 5) are shown. *H,* representative Western blot and quantification of phosphorylated AKT and Cd36 expression in livers from C3H mice subjected to vehicle (*n* = 7) and insulin (*n* = 7) perfusion. *Dotted lines* separate noncontiguous lanes of the same gel. *I,* real time PCR analyses of *Cd36* expression in liver extracts from C3H mice. Data are presented as mean ± S.E., where ***, *p* < 0.001.

In agreement with the notion that insulin enhancement of hepatic Cd36 expression is *Ppar*γ-dependent, exposure of C3H/HeN (C3H) mice, in which hepatic *Ppar*γ expression is deficient (Fig. 6*A*) (21), to the 3w SRD regime did not provoke an increase in hepatic Cd36 mRNA or protein levels (Fig. 6, *B* and *C*). Similarly, hepatic mRNA levels of the *Mogat1* and *Fabp4* were not increased in C3H mice after 3w of SRD (Fig. 6*B*). Moreover, neither PKCϵ membrane translocation nor hepatic TG levels was increased in livers of 3w SRD C3H mice, providing evidence that these mice are protected against developing hepatosteatosis and liver insulin resistance under an SRD regime (Fig. 6, *D* and *E*). Although the normal glucose and insulin levels observed in 3w SRD C3H mice (Fig. 6, *F* and *G*) support the notion that these mice do not develop insulin resistance on an SRD, the normoinsulinemic levels preclude a direct assessment of the role for *Ppar*γ in insulin-stimulated enhancement of hepatic Cd36 expression in C3H mice. Thus, we next perfused livers of C3H mice with insulin. Although insulin potently stimulated Ser^473^ AKT phosphorylation in insulin-perfused C3H livers (Fig. 6*H*), it failed to increase Cd36 protein and mRNA expression (Fig. 6, *H* and *I*).

## Discussion

Hyperinsulinemia is widely considered a consequence of insulin resistance where β-cells, as a compensatory response to the insulin-resistant state, produce and secrete increased levels of insulin, resulting in elevated basal levels of insulin but normoglycemia. Accumulating evidence suggests, however, that hyperinsulinemia and insulin resistance might be partly disconnected and that hyperinsulinemia *per se* is an independent risk factor for the development of perturbed glucose tolerance, T2D and NAFLD (13, 14, 35). Thus, it has been proposed that hyperinsulinemia is not only a consequence of insulin resistance but may also act as an independent driver that triggers, and sustains, insulin resistance and dysglycemia (12). Here, we present *in vivo* and *ex vivo* data providing evidence that increased circulating levels of insulin trigger fatty liver development and insulin resistance by stimulating hepatic expression of the FA transporter Cd36 in a *Ppar*γ-dependent manner. Our study shows that a 3-week SRD is sufficient to provoke hyperinsulinemia, hepatic steatosis, hepatic insulin resistance, and dysglycemia in CBA mice. The increase in insulin levels and hepatic fat accumulation in CBA mice was paralleled by an increase in the hepatic expression of *Ppar*γ and the *Ppar*γ target genes *Cd36, Fabp4,* and *Mogat1*, *i.e.* genes involved in FA uptake and TG synthesis. B6 mice, which are considered to be obesity- and diabetes-prone (36) and therefore extensively used in metabolic studies, showed overall lower basal and glucose-stimulated insulin secretion capacity under metabolically unstressed conditions, *i.e.* on the CD. The reduced insulin secretion potential of B6 mice is a consequence of a deletion in the *nicotinamide nucleotide transhydrogenase* gene (37). Notably, the SRD failed to provoke enhanced insulin levels, hepatic fat accumulation, and dysglycemia in B6 mice, and hepatic expression of *Ppar*γ, *Cd36, Fabp4,* and *Mogat1* was not increased SRD B6 mice. Similarly, C3H mice, which lack *Ppar*γ expression in the liver (21), did not enhance hepatic *Cd36, Fabp4,* and *Mogat1* expression on the SRD diet or in response to liver insulin perfusions, and furthermore, they did not develop hepatosteatosis and hepatic insulin resistance in response to the SRD. These findings not only demonstrate a strong association between hyperinsulinemia, hepatic steatosis, hepatic insulin resistance, and glucose intolerance but also accentuate important metabolic differences between common mouse strains. Despite the elevated basal levels of insulin and higher hepatic TG levels displayed by CD CBA as compared with CD B6 mice, the SRD regime nonetheless triggered hyperinsulinemia in CBA mice, which in turn provoked a significant increase in hepatic TG levels and insulin resistance. Additionally, 3w SRD CBA, but not B6, mice had increased fat mass providing support for the observation that high insulin levels drive obesity under energy-rich conditions (38).

Hepatic Cd36 expression levels are low in normal, nonmetabolically challenged livers but are drastically increased in murine models exposed to a high fat diet and in human NAFLD (6, 7, 17, 39, 40). Moreover, *CD36* expression is increased and positively correlates with plasma insulin levels, insulin resistance, and the degree of steatosis in NAFLD patients (8, 9). Although CD36 is not critically required for hepatic FFA uptake in humans (41), overexpression of *CD36* in human hepatoma cell lines as well as forced expression of *Cd36* in isolated mouse hepatocytes resulted in increased FFA uptake (17, 42). In addition, forced expression of *Cd36* in livers of normal fed, nonmetabolically challenged mice caused hepatic steatosis (10), and an age-related increase in hepatic CD36 expression is associated with increased susceptibility to NAFLD (43). Interestingly, insulin has been shown to enhance Cd36 levels in skeletal and cardiac muscle; under a short term hyperinsulinemic-euglycemic clamp, insulin stimulated Cd36 levels in skeletal muscle, and the increase in Cd36 levels were positively correlated to insulin resistance (44). Insulin has also been shown to directly enhance the expression of Cd36 in isolated rat cardiac myocytes and in perfused intact hearts (45). In agreement with these findings and in support for a potential general role for insulin in stimulating Cd36 expression, we here show that insulin directly enhances Cd36 expression in isolated primary hepatocytes and in a liver perfusion assay. Our data disagree however with the results reported by Aspichueta and co-workers (6) who did not observe increased Cd36 expression in insulin-exposed primary hepatocytes. The use of different concentrations of insulin and different experimental protocols and/or different methodologies used in the two studies may explain this discrepancy.

Pparγ, an upstream positive regulator of *Cd36* expression (16, 17), is also linked to hepatic steatosis. *Ppar*γ, and in particular *Ppar*γ*2*, overexpression promotes hepatic fat accumulation (21–24) in an Srebp-1c independent way (25), whereas a liver-specific deletion of *Ppar*γ attenuates hepatic steatosis, highlighting the important role for *Ppar*γ in fatty liver development (26–28). In agreement with these observations, and in contrast to B6 mice that express *Ppar*γ in the liver, insulin perfusion of C3H livers did not provoke an increase in Cd36 expression. However, we can not exclude that additional strain differences, *i.e.* apart from hepatic *Ppar*γ expression, between B6 and C3H mice contribute to the difference in stimulation of Cd36 expression following liver insulin perfusion. Nonetheless, the failure of insulin to increase *Cd36* expression in primary hepatocytes and HepG2 cells, in which *Ppar*γ had been knocked down, provides direct evidence for a role for *Ppar*γ in mediating the insulin stimulatory effect on *Cd36* expression. The increased expression of *Ppar*γ*2* and its target genes in the livers of obese mouse models and humans with NAFLD (8, 20, 46, 47), *i.e.* under conditions of insulin resistance, might appear contradictory. However, *Ppar*γ expression is in part regulated through a positive feedback loop, and additionally, *Ppar*γ activity is enhanced by ligands, including natural ligands such as fatty acids (48, 49). Thus, our findings together with the observation of increased hepatic *Ppar*γ*2* expression in obese models and NAFLD provide evidence that insulin is sufficient to induce, but is not required for maintenance of, *Ppar*γ*2* expression. Additionally, *Ppar*γ-mediated enhancement of Cd36 expression would increase hepatic uptake of fatty acids, thus providing increased availability of *Ppar*γ ligands that likely further enhance *Ppar*γ activity.

Insulin is known to also stimulate hepatic *Srebp-1c* expression, as well as Srebp-1c activation, thus promoting *de novo* lipogenesis in the liver that in turn likely contributes to fatty liver development in response to energy-rich diets (50, 51). In our experimental model, the expression of *Srebp-1c*, as well as that of downstream lipogenic enzymes, was not enhanced in 3-week SRD-fed CBA mice. Moreover, we did not observe an altered hepatic expression of genes implicated in FA oxidation and the VLDL secretion pathways in 3w SRD CBA mice. Together, these findings argue against *de novo* lipogenesis, decreased FA oxidation, or reduced VLDL secretion as major contributors to the fat accumulation and hepatosteatosis observed in 3w SRD CBA mice. However, these data do not exclude that these pathways contribute to fatty liver development and that increased expression of genes controlling these pathways would become prominent under other experimental conditions, including a prolonged SRD regime.

In summary, we show that increased intake of sucrose in CBA mice results in rapid development of hyperinsulinemia, hepatosteatosis, and insulin resistance and that insulin enhances hepatic expression of the FA transporter Cd36 in a *Ppar*γ-dependent manner. Thus, our data suggest that diet-induced hyperinsulinemia is an early and potent inducer of hepatosteatosis, insulin resistance, and dysglycemia that may predispose individuals to T2D and NAFLD. Notably, a relatively recent prospective study involving a 24-year follow up of 515 individuals identified basal hyperinsulinemia in normoglycemic individuals as an independent risk factor for the development of dysglycemia (35). In conclusion, our observations support the notion of hyperinsulinemia as an initiator and driver of insulin resistance and dysglycemia. Additionally, our findings reinforce the view that determination of basal insulin levels may be clinically relevant for diagnosing early dysglycemia. Monitoring of insulin levels combined with therapeutic intervention and life style changes aimed at restoring normoinsulinemia in insulin-resistant individuals would likely help to impede a further deterioration of insulin sensitivity and glucose homeostasis and thus the development of overt T2D and NAFLD.

## Author Contributions

P. S. and A. S. contributed to the design and performance of experiments, data interpretation, results, and writing and editing of the manuscript. F. B., J. S., and I. S. contributed to the performance of experiments. H. E. designed and supervised the study, analyzed and interpreted the data, and wrote the paper. All the authors approved the final version of the manuscript. H. E. is responsible for the integrity of the work as a whole.

## Footnotes

2 The abbreviations used are:

T2D: Type 2 diabetes
NAFLD: nonalcoholic fatty liver disease
SRD: sucrose-enriched diet
PPAR: peroxisome proliferator-activated receptor
FFA: free fatty acid
FA: fatty acid
GSIS: glucose-stimulated insulin secretion
GTT: glucose tolerance test
ITT: insulin tolerance test
qRT: quantitative RT
CD: Control Diet
3w: 3 weeks
TG: triglyceride
HOMA-IR: homeostasis model for insulin resistance.
